# Racial and ethnic disparities in postnatal growth among very low birth weight infants in California

**DOI:** 10.1038/s41372-023-01612-9

**Published:** 2023-02-03

**Authors:** Soon Min Lee, Lillian Sie, Jessica Liu, Jochen Profit, Elliot Main, Henry C. Lee

**Affiliations:** 1 Department of Pediatrics, Division of Neonatal & Developmental Medicine, Stanford University, Stanford, CA USA; 2 California Perinatal Quality Care Collaborative, Stanford, CA USA; 3 Department of Pediatrics, Yonsei University College of Medicine, Seoul, South Korea; 4 Department of Obstetrics, Stanford University, Stanford, CA USA; 5 California Maternal Quality Care Collaborative, Stanford, CA USA; 6 Department of Pediatrics, Division of Neonatology, University of California San Diego, San Diego, CA USA

**Keywords:** Epidemiology, Outcomes research

## Abstract

**Objective:**

To identify racial/ethnic disparities in postnatal growth by year and gestational age among very low birth weight infants.

**Study design:**

Total 37,122 infants, with birth weight 500–1500 g or gestational age 23–34 weeks in the California Perinatal Quality Care Collaborative in 2008–2016. Postnatal growth failure (PGF) was defined as change in weight Z-score from birth to discharge below −1.28. Multivariable regression analysis with birth hospital as random effect was used to estimate odds ratios (OR).

**Results:**

Infants born to Hispanic mothers had highest risk of PGF at 30%, compared to white (24%, OR 1.33), Black (22%, OR 1.50), or Asian/Pacific Islander mothers (23%, OR 1.38). PGF incidence decreased from 2008 (27.4%) to 2016 (22.8%) with differences in trends by race. Each increasing gestational age week was associated with decreasing risk for PGF (OR 0.73, 95% confidence interval 0.72–0.74).

**Conclusion:**

Targeted interventions addressing PGF are needed to address disparities.

## Introduction

Although the survival of preterm infants has improved markedly, postnatal growth remains a prominent concern for this population because of its link to brain growth and neurodevelopment. Postnatal growth among very low birth weight (VLBW) infants is influenced by a variety of factors, such as neonatal morbidities, nutrition-related factors, sex, and small for gestational age (SGA) (1, 2). However, less is known about racial/ethnic disparities in postnatal growth in preterm infants.

Measures of postnatal growth failure (PGF) for preterm infants have varied among studies [1–4]. In the 1990s, more than 95% of VLBW infants were discharged below the 10th percentile of weight-for-age [5, 6]. In a quality improvement collaborative sponsored by the Vermont Oxford Network, the development and implementation of evidence-based better nutrition support practices in preterm infants led to improved growth at the time of discharge. [7] Among VLBW infants in the Vermont Oxford Network, PGF declined by 14% and severe PGF by 12% from 2000 to 2013 [3]. In the California Perinatal Quality Care Collaborative (CPQCC), the adjusted fall in weight Z-score between birth and discharge decreased significantly between 2005 and 2012 by 0.016 Z-scores/year, and growth failure as defined as a weight Z-score decrease of more than 1.0 during admission fell from 47% in 2005 to 38% in 2012 [2]. CPQCC includes growth velocity as 1 of 9 key measures in the Baby-MONITOR, a composite indicator of neonatal intensive care unit (NICU) quality [8]. Several quality improvement collaboratives have focused their efforts on improving nutrition and growth [9–11]. A second major quality improvement project from CPQCC was launched to optimize the growth and nutrition of VLBW infants in 2018. However, the effects of race/ethnicity on postnatal growth were not reported.

Disparities in fetal growth restriction, infant postnatal growth rates, and nutrition practice such as breast milk feeding exist by race and ethnicity [12–15]. Offspring of Black versus white women had a 78% greater risk of SGA [16]. In a study from Sweden, SGA was more likely based on the mother and/or father having Black or Asian race [17]. A study about racial disparities in weight gain velocity and body composition showed Black preterm infants had higher weight gain from birth to discharge, but comparable body composition measurements at discharge [18]. Being born to an Asian mother has been associated with a deceleration in growth compared to infants born to white mothers [19]. Without more granular knowledge of growth patterns, it is challenging to evaluate and target preterm infants’ race/ethnicity-specific growth trajectories. Understanding racial/ethnic disparities in postnatal growth is important to address long-term inequities in health and development.

Our objective was to investigate racial/ethnic differences in PGF among VLBW infants in California. We specifically focused on longitudinal trends in growth and factors associated with PGF.

## Methods

### Subject population

Data were extracted from the CPQCC database, which at the time of the study period included data collected across 132 NICUs that accounted for the care of more than 95% of VLBW infants born in California. Data from California’s Department of Health Care Access and Information were linked to CPQCC records to obtain detailed maternal information, including race, ethnicity, maternal education, and payer type.

We included 37,122 infants who were born with a birth weight of 500–1500 g or with gestational age of 23–34 weeks from 2008 to 2016 and discharged alive before 50 weeks corrected gestational age. The limit of 50 weeks corrected gestational age was chosen to exclude those patients with special circumstances leading to longer hospitalization; the Fenton growth charts are available up to 50 weeks postmenstrual age (PMA). A subset of 20,019 infants born with a birth weight below 1000 g or gestational age of 23–28 weeks were selected for subgroup analysis. Infants with severe congenital anomalies were excluded, as were patients with missing data for birth weight, discharge weight, or sex. In addition, we excluded patients with a weight Z-score at birth or discharge more than five standard deviations above or below the expected mean as an error. The Institutional Review Board at Stanford University approved the study.

### Outcomes and variables

Trained data abstractors prospectively gathered maternal, delivery, and neonatal data based on standardized definitions from the CPQCC manual of operations. Variable definitions align with those developed by the Vermont Oxford Network. Gestational age was determined by the best available estimate in weeks and days. Where sources disagreed, obstetric measures based on the last menstrual period or prenatal ultrasound took precedence over the neonatologist’s estimate based on physical examination. Weight was measured and recorded at birth and discharge. To control for variation in gestational age and postnatal age, body weight was converted into a Z-score using the provided calculator of the Fenton growth chart that can be downloaded at https://www.ucalgary.ca/fenton/2013chart/. [20, 21]. SGA was defined as weight Z-score at birth and discharge below −1.28 (equal to 10th percentile for age). To ascertain the degree of postnatal growth, the Z-score at birth was subtracted from the Z-score at discharge. PGF was defined as a change in weight Z-score from birth to discharge below −1.28 (equal to 10th percentile) using the Fenton growth charts. A weight Z-score at birth and discharge below the 3rd percentile was indicated by Z < −2. Maternal race/ethnicity was first collected from the data from CPQCC and then Department of Health Care Information/Vital Statistics if data from CPQCC were not available. Data collection for maternal race in CPQCC includes the following categories: White, Black, Asian American/Pacific Islander (Asian/PI; this group includes Native Hawaiian), American Indian/Alaska Native, Other, and Unknown. Ethnicity is categorized as Hispanic or non-Hispanic. In our data, Hispanic individuals are most likely to have race category chosen as white, unknown, or other in somewhat equal numbers, and a very small proportion picking other categories of race. We combined all race categories as Hispanic for those with Hispanic ethnicity. We did not have the ability to consider those who may identify as more than one race due to limitations of data collection during the study period. White race was used as the reference group due to this group known to have higher quality care and outcomes potentially due to structural racism, and other groups having minoritized status in our society.

Feeding data were collected by choosing formula only or human milk only or human milk in combination with either fortifier or formula. Formula only indicated that the infant was discharged receiving formula milk as their only enteral feeding. Outborn was defined as the infant being born in another facility or at any location outside the enrolled center or home at any time after birth. Hypertension was defined according to the maternal or infant medical record stating the diagnosis of hypertension, chronic or pregnancy-induced, eclampsia, preeclampsia, seizures, toxemia, HELLP syndrome, with or without edema and proteinuria, or if a maternal blood pressure above 140 systolic or 90 diastolic was recorded before or during the current pregnancy. Chorioamnionitis was defined as the maternal medical record noting evidence of infection of the amniotic sac and fluid or infection of the uterine wall. CLD was defined as oxygen use at 36 weeks postmenstrual age; periventricular leukomalacia (PVL) as evidence of cystic PVL on cranial imaging at any time; necrotizing enterocolitis as clinical and radiographic findings stage 2 or 3; severe intraventricular hemorrhage (IVH) as grade 3 or 4 IVH by cranial imaging obtained before 28 days after delivery; severe retinopathy of prematurity as ≥ stage 3 disease or treatment with retinal ablation surgery or anti-vascular endothelial growth factor drug.

### Statistical analysis

We used one-way ANOVA and χ^2^ tests to examine bivariate associations between characteristics and race/ethnicity. Linear regression models determined if the change in the incidence of PGF was significant over time and by gestational age, stratified by race/ethnicity. Multivariable logistic regression models with hospital accounted for as a random effect were used to examine risk factors associated with growth failure. Variables in the model included race/ethnicity, sex, gravidity, maternal education, maternal age, maternal insurance, SGA at birth, maternal hypertension, chorioamnionitis, CLD, gastrointestinal perforation, necrotizing enterocolitis, late-onset sepsis, with birth hospital applied as a random effect to adjust for clustering by site. Length of stay was highly correlated with included morbidities and was therefore not included as a covariate. All statistical analyses were performed using SAS version 9.4 (SAS Institute, Cary, North Carolina). *P*-values < 0.05 were considered statistically significant.

## Results

Among 37,122 infants, the distribution of maternal race/ethnicity was 47% Hispanic, 26% white, 13% Black, 11% Asian/PI, and 3% other. For the 20,019 infants with birth weight below 1000 g or having gestational age of 23–28 weeks, the distribution of maternal race/ethnicity was 49% Hispanic, 25% white, 13% Black, 10% Asian/PI, and 3% other.

**Fig. 1:**
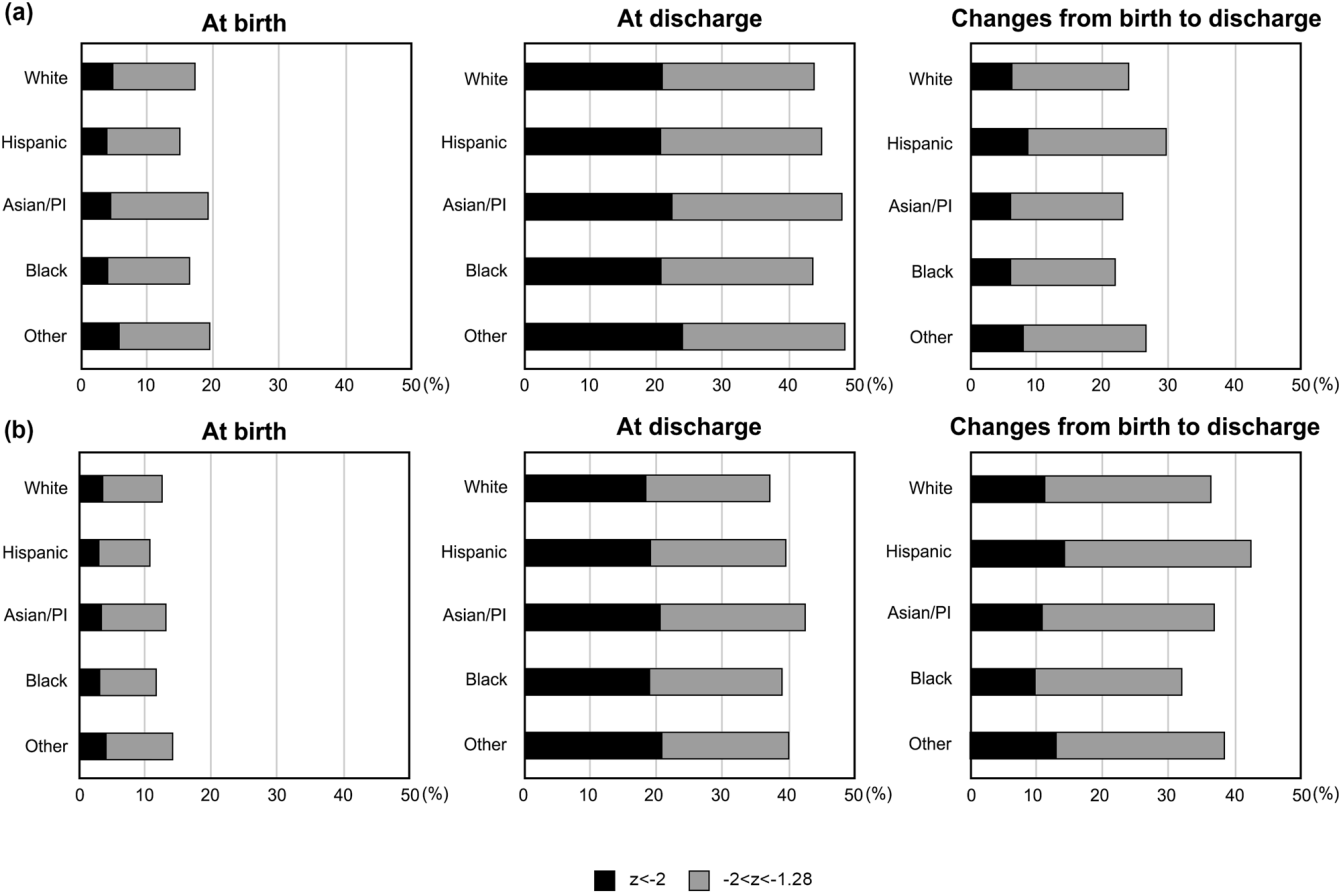
Incidence of infants with weight z-score below −1.28 at birth and discharge and changes in weight z-score between birth and discharge below −1.28 by race/ethnicity among. **a** infants 23–34 weeks of gestation or <1500 g and **b** infants 23–28 weeks of gestation or <1000 g [**a** Total 37 122 infants with Hispanic 17,404, white 9656, black 4652, Asian/PI 4223, other 1187, **b** Total 20,019 infants with Hispanic 9809, white 5005, black 2602, Asian/PI 2002, other 601].

Table 1 shows the patients’ demographic characteristics and morbidities by maternal race/ethnicity. Mean z-scores at birth and discharge were both lowest in Asian/PI infants. However, changes in weight Z-score between birth and discharge was largest in Hispanic infants. The incidence of SGA status (Z-score < −1.28) at birth and at discharge was more common in white or Asian/PI infants than Hispanic infants. However, incidence of PGF (change in weight Z-score from birth to discharge below −1.28 was more common in Hispanic infants (Fig. 1). Infants born to Hispanic mothers had highest risk of PGF at 30%, compared to white (24%), Black (22%), or Asian/Pacific Islander mothers (23%).

**Table 1:** Demographic characteristics by maternal race/ethnicity.

	Hispanic (17,404)	White (9656)	Black (4652)	Asian/PI (4223)	Other (1187)	*P*-value
Gestational age, mean (SD)	28.3 (2.6)	28.7 (2. 6)	28.3 (2.7)	28.8 (2.6)	28.7(2.6)	<0.001
Z-score at birth, mean (SD)	−0.2 (1.0)	−0.3 (1.0)	−0.4 (0.9)	−0.4 (0.9)	−0.4 (1.0)	<0.001
Z-score at discharge, mean (SD)	−1.2 (1.0)	−1.2 (1.0)	1.2 (1.0)	−1.3 (1.0)	−1.3 (1.0)	<0.001
Changes in Z-score, mean (SD)	−1.0 (0.8)	−0.9 (0.7)	−0.8 (0.7)	−0.9 (−0.7)	−0.9 (0.7)	<0.001
Male, *n* (%)	9054 (51)	4953 (50)	2276 (48)	2188 (51)	606 (50)	0.048
Multiple pregnancy, *n* (%)	3645 (21)	3644 (38)	1133 (24)	1325(31)	347 (29)	<0.001
SGA at birth, *n* (%)	2848 (16)	1797 (18)	844 (18)	894 (21)	251 (21)	<0.001
Nulliparous, *n* (%)	3955 (40)	2484 (46)	1102 (41)	962 (44)	258 (43)	<0.001
Maternal age < 20, *n* (%)	1801 (11)	411 (5)	429 (10)	86 (2)	44 (4)	<0.001
20–34, *n* (%)	11,346 (69)	5885 (66)	3134 (71)	2171 (58)	728 (67)	
35–39, *n* (%)	2357 (14)	1805 (20)	631 (14)	1067 (28)	212 (20)	
≥40, *n* (%)	917 (5)	805 (9)	232 (5)	455 (2)	98 (9)	
Maternal education College or more, *n* (%)	3054 (32)	3632 (71)	1319 (52)	1610 (80)	395 (71)	<0.001
High school, *n* (%)	2924 (31)	1078 (21)	814 (32)	316 (16)	120 (22)	
Some HS or less, *n* (%)	3620 (38)	375 (7)	430 (17)	99 (5)	44 (8)	
Insurance public, *n* (%)	6443 (37)	3713 (38)	952 (20)	1642 (38)	348 (29)	<0.001
Private, *n* (%)	3158 (18)	1505 (15)	1622 (34)	476 (11)	210 (17)	
other, *n* (%)	8023 (46)	4563 (47)	2139 (45)	2191 (51)	649 (54)	
Gestational hypertension^a^, *n* (%)	263 (2)	153 (2)	59 (1)	67 (2)	14 (1)	0.518
Chorioamnionitis, *n* (%)	1067 (6)	561 (6)	486 (10)	228 (5)	70 (6)	<0.001
Gestational DM, *n* (%)	1251 (7)	555 (6)	231 (5)	496 (12)	90 (8)	<0.001
Hospital transfer, *n* (%)	9007 (52)	4826 (51)	2548 (55)	1948 (46)	621 (53)	<0.001
Early sepsis, *n* (%)	223 (1)	84 (1)	47 (1)	42 (1)	10 (1)	0.022
Respiratory distress syndrome, *n* (%)	11,893 (68)	6851 (71)	3096 (67)	2735 (65)	771 (65)	<0.001
Late sepsis *n* (%)	1030 (6)	401 (4)	265 (6)	188 (5)	55 (5)	<0.001
Necrotizing enterocolitis^b^, *n* (%)	561 (3)	244 (3)	168 (4)	100 (2)	33 (3)	0.002
Gastrointestinal perforation *n* (%)	330 (2)	126 (1)	66 (1)	61 (2)	20 (2)	0.002
Chronic lung disease, *n* (%)	3694 (23)	1991 (22)	917 (21)	832 (21)	257 (23)	0.006
Severe ROP^c^, *n* (%)	892 (7)	375 (5)	200 (6)	227 (7)	64 (7)	<0.001
Severe IVH^d^, *n* (%)	830 (5)	968 (4)	231 (5)	119 (3)	32 (3)	<0.001
PVL, *n* (%)	334 (2)	166 (2)	94 (2)	74 (2)	19 (2)	0.549
Formula feeding only, *n* (%)	5194 (33)	2409 (27)	2034 (48)	808 (20)	326 (29)	<0.001

*DM* Diabetic Mellitus, *IVH* intraventricular hemorrhage, *PVL* Periventricular leukomalacia, *ROP* retinopathy of prematurity, *SGA* small for gestational age.

^a^defined as if the maternal or infant medical record states the diagnosis of hypertension, chronic or pregnancy-induced, eclampsia, preeclampsia, seizures, toxemia, HELLP syndrome, with or without edema and proteinuria, or if a maternal blood pressure above 140 systolic or 90 diastolic was recorded before or during the current pregnancy,

^b^defined as clinical and radiographic findings stage 2 or 3,

^c^severe ROP as ≥ stage 3 disease or treatment with retinal ablation surgery or anti-vascular endothelial growth factor drug,

^d^severe IVH as grade 3 or 4 IVH by cranial imaging obtained before 28 days after delivery;

Hispanic infants were at higher risk of PGF than white infants (odds ratio [OR], 1.33; 95% confidence interval [CI], 1.26–1.41; *P* < 0.0001). Black infants were at lower risk of PGF compared to white infants (OR, 0.89; 95% CI: 0.82–0.96; *P* = 0.0045). Asian/PI infants were not significantly different from white infants. The subgroup of infants born at <1000 g or at 23–28 weeks of gestation exhibited a similar pattern of SGA at birth and discharge and PGF as the larger cohort according to race/ethnicity; however, a lower incidence of SGA at birth and higher incidence of PGF was observed compared to the full cohort.

**Fig. 2:**
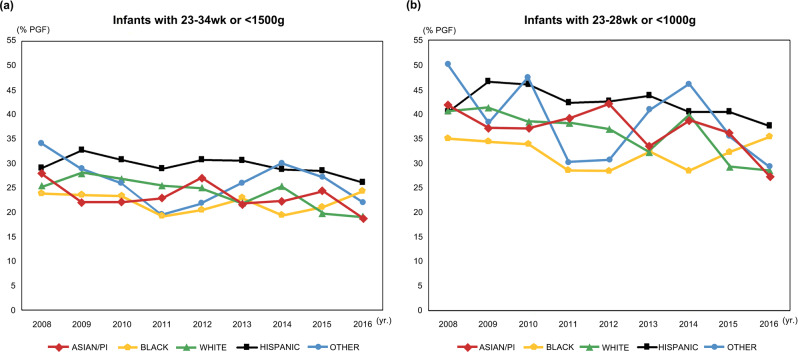
Incidence of growth failure by year according to race/ethnicity among. **a** infants 23–34 weeks of gestation or <1500 g and **b** subgroup of infants 23–28 weeks of gestation or < 1000 g [**a** Total 37,122 infants with Hispanic 17,404, white 9656, black 4652, Asian/PI 4223, other 187, **b** Total 20,019 infants with Hispanic 9809, white 5005, Black 2602, Asian/PI 2002, other 601].

The incidence of PGF decreased from 2008 (27%) to 2016 (23%) (*P* < 0.0001). Serial PGF trends over time differed significantly by race (*P* < 0.0001); infants born to Hispanic mothers were at higher risk of PGF than those born to white (OR, 1.33; 95% CI: 1.25–1.41) or Black (OR, 1.50; 95% CI: 1.39–1.62) or Asian/PI mothers (OR, 1.38; 95% CI: 1.28–1.49) (Fig. 2a). For subgroup of infants < 1000 g or 23–28 weeks gestation, the incidence of PGF according to race/ethnicity by year showed significant difference with gradual improvement (Fig. 2b).

**Fig. 3:**
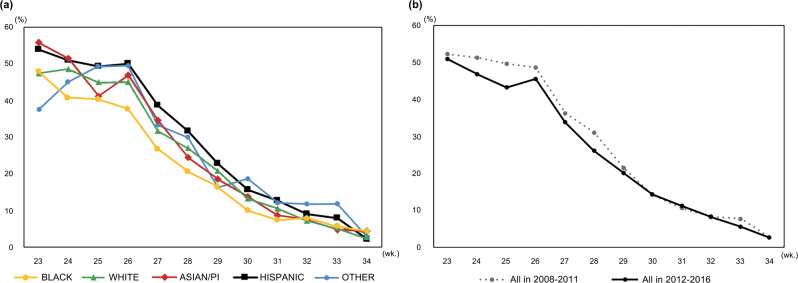
Incidence of growth failure by gestational age. **a** according to race/ethnicity, and **b** comparing 2008–2011 to 2012–2016 among total 37,122 infants.

The incidence of PGF decreased with increasing gestational age (from 52% of 23 weeks’ gestation to 3% of 34 weeks’ gestation) (*P* < 0.0001). Each week of gestational age was associated with decreasing risk for PGF (OR 0.73, 95% CI 0.72–0.74, *P* < 0.0001). For Hispanic infants, those between 25 and 33 weeks’ gestation showed the highest incidence of PGF. For Asian/PI infants, those between 23 and 24 weeks of gestation showed the highest incidence of PGF (Fig. 3a). When comparing the incidence of PGF between 2012 and 2016 with incidence of PGF between 2008 and 2011, significant improvements in decreasing PGF was found overall (*P* < 0.0001), and especially for infants born below 29 weeks’ gestation (Fig. 3b).

In multivariable analysis, race/ethnicity in addition to the various morbidities, sex, formula feeding only, maternal hypertension, and SGA at birth were independently associated with PGF. Hispanic ethnicity was associated with higher risk of PGF, while Black race reduced the risk of PGF (Table 2). Birth hospital, included as a random effect in the model, was a significant factor influencing PGF (Z = 7.64, *P* < 0.0001). Maternal age, insurance type, maternal education, multiple pregnancy, maternal chorioamnionitis, early sepsis, and PVL did not significantly influence PGF rates. As for the subgroup below 1000 g or 28 weeks of gestation, race/ethnicity, sex, formula feeding only, and maternal hypertension were associated with PGF. Hispanic ethnicity was associated with increased risk of PGF, while Black race was associated with reduced risk (Table 2). Birth hospital was a significant risk factor for PGF (Z = 7.64, *P* < 0.0001). Maternal age, maternal education, SGA at birth, maternal chorioamnionitis, early onset sepsis, severe IVH, PVL, and CLD were not associated with PGF.

**Table 2:** Risk factors for postnatal growth failure^a^ among Infant with 23–34 week of gestation or <1500 g and Infant with 23–28 week of gestation or <1000 g using logistic regression considering birth hospital as random effect.

Infant with 23–34 week of gestation or <1500 g			Infant with 23–28 week of gestation or <1000 g		
	Adjusted OR^b^ (95% CI)	*P*-value		Adjusted OR^b^ (95% CI)	*P*-value
Race		<0.0001	Race		<0.0001
Asian/PI vs White	0.91 (0.76–1.09)	0.23	Asian/PI vs White	1.04 (0.83–1.30)	0.19
Black vs White	0.71 (0.60–0.84)	0.002	Black vs White	0.66 (0.54–0.80)	0.004
Hispanic vs White	1.13 (1.00–1.27)	0.03	Hispanic vs White	1.08 (0.93–1.26)	0.009
other vs white	1.08 (0.81–1.43)	0.57	Other vs white	1.07 (0.75–1.52)	0.66
Male vs female	0.77 (0.70–0.84)	<0.0001	Male vs female	0.82 (0.73–0.91)	0.0002
Maternal hypertension	0.56 (0.50–0.63)	<0.0001	Maternal hypertension	0.60 (0.52–0.68)	<0.0001
Formula feeding only	0.81 (0.74–0.90)	<0.0001	Formula feeding only	0.75 (0.67–0.84)	<0.0001
Respiratory distress	1.74 (1.54–1.97)	<0.0001	Respiratory distress	1.28 (1.08–1.51)	0.0041
Necrotizing enterocolitis	2.57 (2.05–3.22)	<0.0001	Necrotizing enterocolitis	2.42 (1.87–3.13)	<0.0001
Gastrointestinal perforation	2.62 (1.86–3.70)	<0.0001	Gastrointestinal perforation	2.52 (1.76–3.61)	<0.0001
PDA	2.06 (1.86–2.27)	<0.0001	PDA	1.59 (1.41–2.01)	<0.0001
Late onset sepsis	1.61 (1.37–1.90)	<0.0001	Late onset sepsis	1.45 (1.21–1.74)	<0.0001
Severe ROP	1.96 (1.65–2.32)	<0.0001	Severe ROP	1.68 (1.41–2.00)	<0.0001
SGA at birth	1.19 (1.03–1.38)	0.02			
Severe IVH	1.33 (1.09–1.63)	0.005			
Chronic lung disease	1.19 (1.06–1.32)	0.002			

*IVH* intraventricular hemorrhage, *PDA* patent ductus arteriosus, *ROP* retinopathy of prematurity.

^a^Postnatal Growth Failure was defined as a change in weight Z-score from birth to discharge below −1.28 using the Fenton growth charts.

^b^Variables in the model included race/ethnicity, sex, gravidity, maternal education, maternal age, maternal insurance, SGA at birth, maternal hypertension, chorioamnionitis, CLD, gastrointestinal perforation, late-onset sepsis, with birth hospital applied as a random effect to adjust for clustering by hospital.

## Discussion

In this large population-based cohort of VLBW infants born in California, we demonstrated that race/ethnicity was associated with PGF. SGA at birth and discharge was highest among infants of Asian/PI mothers; however, the incidence of PGF was highest in Hispanic infants. Racial/ethnic disparities in postnatal growth among VLBW infants may have implications for careful monitoring and attention to nutrition.

Being born SGA is a predictor of failure to grow and careful monitoring of nutrition and personalized strategies may be needed to improve outcomes. Racial/ethnic disparities in SGA at birth have been studied. Mexican American infants tended to have higher weight compared with non-Hispanic white infants between 30 and 37 weeks of gestation [22]. Other studies have shown that rates of low birth weight and SGA were elevated among Asian Indians compared to white infants [23–25]. Our study extends these findings to VLBW infants demonstrating the highest rate of SGA among Asian/PI infants compared to white, Black, Hispanic, and other infants, and the lowest rates of SGA among Hispanic infants.

If growth failure is determined based only on the percentage of body weight at discharge, SGA status at birth may have a disproportionate impact compared to other factors, and differences by gestational age and sex may be overlooked. In this study, among 6634 SGA infants, 96% remained SGA at discharge; however, only 29% noted SGA at discharge among 37,122 AGA infants. In this study, among infants between 23 to 28 weeks of gestation or <1000 g, SGA at birth was not significantly associated with PGF when considered in multivariable logistic regression with birth hospital accounted for as a random effect.

The change in Z-score between birth and discharge has been used for evaluation of growth. We have similarly defined PGF as changes of weight z-score from birth to discharge below −1.28 (equal to the 10th percentile) and confirmed PGF was shown in 39% of the infant. We also found that Hispanic infants, while having a low incidence of SGA at birth, had the highest incidence of PGF in California NICUs. Hispanic ethnicity was an independent risk factor (OR 1.15) for PGF, adjusted for comorbidities and socioeconomic status.

Although human milk is the preferred feeding choice for its known impact on prevention of necrotizing enterocolitis and its association with other short- and long-term clinical benefits, formula feeding at discharge has been associated with higher growth velocity [26, 27]. A prior study using 2008–2011 data from the CPQCC showed that Hispanic infants had higher rates of breast milk at discharge, particularly when the mother was foreign-born [28]. We also confirmed formula feeding only at discharge was associated with lower incidence of PGF (OR 0.81).

PGF has been associated with various factors including nutrition, morbidities, sex, and SGA. In other studies, the comorbidities most associated with poorer postnatal growth included IVH, retinopathy of prematurity, necrotizing enterocolitis, isolated gastrointestinal perforation, and CLD [29, 30]. Our results confirm prior findings and add new knowledge regarding maternal factors associated with PGF. We found that infants with maternal hypertension and SGA at birth were at an increased risk for PGF. These findings are consistent with previous study relating gestational hypertension and SGA to discharge weight, length, and head circumference being below the 10th percentile [31]. An important driver of preterm birth is hypertensive disorders including preeclampsia, which may lead to maternal vasoconstriction, insufficient placental blood flow, low fetal nutritional reserve, and intrauterine growth restriction. While not included in our analysis, pre-pregnancy body mass index may be a modifiable intrauterine exposure that influences infant postnatal anthropometric outcomes within first 6 months of life [32].

Despite intensive treatment, chronic nutritional deprivation and growth failure in preterm infants continue to be challenging in the NICU. About a 10% (from 65 to 58%) decrease was noted during the early 2000s, with subsequent smaller incremental improvement from 55 to 50% between 2008 and 2013 [3]. Statewide collaborative quality improvement in New York achieved significant improvement, with a 0.06% decrease in the difference in weight Z-scores between birth and discharge with improvement noted in 78% of centers during a 48-month-long project [9]. In the current study, although not directly associated with a targeted QI project for improving postnatal growth, PGF decreased from 29 to 22% and the fall in weight Z-score between birth and discharge decreased by 0.06% between 2008 and 2016. During the study period, activities in CPQCC included implementation of a breast milk/nutrition change package to increase breast milk feeding and inclusion of growth velocity as 1 of 9 keys in a composite indicator of NICU quality [8, 33]. In our analysis, birth hospital was confirmed as a significant predictor of growth failure (Z = 7.64, *P* < 0.0001), highlighting variation across hospitals. Collaborative quality improvement initiatives supporting postnatal growth, such as the CPQCC Grow Baby Grow Collaborative, which was launched in 2018 to optimize the growth and nutrition of VLBW infants to reduce growth failure at discharge, may help mitigate hospital-level effects.

A strength of our study is the database including a large diverse population that covers over 95% of VLBW infants cared for in California. Given the varied population and care settings, our findings may hold lessons for other states. Our study has clinical relevance given the established association of PGF with adverse neurodevelopmental outcomes [34, 35]. Interventions that address PGF may help to decrease racial and ethnic disparities in long-term outcomes. Optimizing nutrition for and careful monitoring of infants born to Hispanic mothers as well as efforts to reduce comorbidities are worthwhile intervention goals.

This study had several limitations, including lack of quantitative nutritional data, monitoring of growth at only birth and discharge, and lack of assessment of other parameters of growth, including length and head circumference. Formula or human milk feeding data were collected only at discharge. Maternal smoking, maternal obesity, and maternal body mass index as potential covariates were not examined. Because infants who died before discharge or were discharged after 51 weeks postmenstrual age were excluded, the possibility of survivor bias and selection bias may exist. An association should not be interpreted as causal, given potential confounding from unobserved variables.

## Conclusions

We found racial and ethnic disparities among Hispanic, Black, Asian/PI, and white VLBW infants in early postnatal growth. Interventions implemented early in life to target this risk factor could help improve both short- and long-term outcome, particularly for Hispanic infants.

## Data Availability

The data for this study are protected under data use agreements with participating hospitals. Specific elements may be available upon reasonable request.
